# Comparative evaluation of flexible ureterorenoscopy and mini-percutaneous nephrolithotomy for the management of renal pelvic stones less than 2 cm with high attenuation value in pediatric patients: a prospective randomized study

**DOI:** 10.1007/s00240-026-01938-x

**Published:** 2026-02-03

**Authors:** Ossama Mahmoud, Mahmoud Abdallah, Ahmed Abdellatif, Ahmed Hamdy Mostafa, Anas Albudairat, Akram A. Elmarakbi, Hany F. Badawy

**Affiliations:** 1 Urology Department, Faculty of Medicine, Beni-Suef University, Beni-Suef, Egypt; 2 Urology Department, Alexandria Police Hospital, Alexandria, Egypt; 3 Urology Department, Alkhor Hospital, Hamad Medical Corporation, Qatar, Egypt

**Keywords:** Retrograde intrarenal surgery, Mini-percutaneous nephrolithotomy, Pediatric renal stones, Stone-free rate, Perioperative outcomes

## Abstract

This study aimed to evaluate the effectiveness and safety of retrograde intrarenal surgery (RIRS) compared with mini-percutaneous nephrolithotomy (mini-PCNL) in children with renal pelvic stones < 2 cm and density > 1000 HU. A prospective randomized clinical trial was conducted at Beni-Suef University Hospitals including 70 children aged 3–14 years. Eligible participants had a single renal pelvic stone < 2 cm with density > 1000 HU on low-dose non-contrast CT and were randomized equally to undergo RIRS or mini-PCNL (*n* = 35 each). The stone-free rates at one month were 91.4% for RIRS and 94.3% for mini-PCNL (*p* = 0.643). RIRS was associated with shorter operative time (72.7 vs. 80.5 min), reduced hospital stay (1.1 vs. 1.3 days), shorter catheterization (1.2 vs. 2.1 days), and lower radiation exposure (34.6 vs. 115.1 s) (all *p* < 0.05). Hemoglobin drop was minimal and not significant in RIRS (0.01 g/dL; *p* = 0.097) and greater in mini-PCNL (0.74 g/dL; *p* < 0.001). Complication rates were similar (11.4% vs. 17.1%; *p* = 0.550). Both RIRS and mini-PCNL are effective and safe for managing pediatric renal stones < 2 cm with high density. RIRS offers comparable efficacy with less invasiveness, shorter recovery, and lower radiation exposure. Mini-PCNL remains a valuable option in anatomically complex or lower-calyceal cases.

**Supplementary Information:**

The online version contains supplementary material available at 10.1007/s00240-026-01938-x.

## Introduction

The overall lifetime prevalence of nephrolithiasis is estimated to affect approximately 14% of the global population. Although the incidence in pediatric patients remains proportionally lower than in adults, a notable rise in diagnosed cases has been reported over the past decades [1]. Pediatric urinary stone disease often presents a diagnostic challenge due to its variable and age-dependent clinical presentation. While infants may exhibit non-specific symptoms such as irritability, vomiting, and inconsolable crying, older children commonly present with flank pain, hematuria, or signs of recurrent urinary tract infections. If left untreated, pediatric urinary stones may lead to severe pain, urinary tract infections, hematuria, and in more advanced cases, renal function impairment [2].

Therefore, timely and effective intervention is essential to minimize morbidity and preserve renal function. Current interventional strategies are largely guided by stone size and location. According to the European Association of Urology (EAU) guidelines, extracorporeal shockwave lithotripsy (ESWL) is recommended as a first-line treatment for renal stones < 20 mm due to its non-invasive nature [3]. However, ESWL is associated with several limitations, including the need for multiple sessions, lower stone-free rates (SFR of 43.8%–82.4%), and reduced efficacy in stones > 10 mm, stones with Hounsfield units > 1000 (e.g., calcium oxalate monohydrate or cystine), and in patients with complex anatomy [4].

The emergence of minimally invasive techniques such as retrograde intrarenal surgery (RIRS) and percutaneous nephrolithotomy (PCNL) has transformed pediatric stone management [5]. Given children’s smaller renal anatomy and reduced surgical tolerance, the application of adult-sized instruments can increase the risk of iatrogenic injury. Miniaturized PCNL (mini-PCNL) was introduced as a modification of standard PCNL to reduce tract size and associated complications. It has since gained popularity in pediatric endourology, with reported SFRs ranging from 70.1% to 97.3% after a single session [6, 7].

In parallel, flexible ureteroscopy lithotripsy has become increasingly utilized in pediatric populations due to its natural access through the urinary tract, reduced trauma, and fast recovery. Compared to PCNL, it offers excellent safety and efficacy, with reported SFRs between 76% and 100%, low retreatment rates (0%–19%), and complication rates ranging from 0% to 28% [8]. Additionally, it allows complete access to the renal collecting system, including challenging locations such as the lower pole calyx [9]. We hypothesized that RIRS would achieve comparable stone-free rates with more favorable perioperative outcomes compared to mini-PCNL. Given these evolving options, this study aims to compare the efficacy and safety of RIRS and mini-PCNL in managing renal pelvic stone < 2 cm with density above 1000 HU in pediatric patients.

## Patients and methods

### Study design

**Fig. 1:**
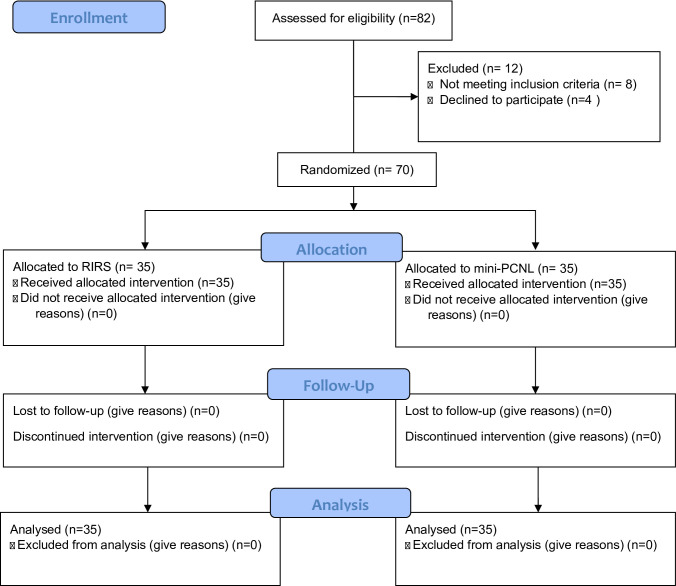
CONSORT flow diagram of patient enrollment and allocation

This was a two-arm, parallel-group, superiority randomized controlled trial with a 1:1 allocation ratio conducted at Beni-Suef University Hospitals, including 70 pediatric patients with renal pelvic stones < 2 cm in diameter and Hounsfield units > 1000 on preoperative low-dose non-contrast computed tomography (low-dose NCCT). This superiority design was chosen to detect clinically meaningful differences between the two interventions (RIRS and mini-PCNL) in terms of effectiveness and safety outcomes, rather than to simply demonstrate equivalence or non-inferiority. Patients were randomly allocated into two equal groups: 35 patients underwent mini-percutaneous nephrolithotomy (mini-PCNL) and 35 underwent retrograde intrarenal surgery (RIRS). Ethical approval was obtained from the Faculty of Medicine, Beni-Suef University Research Ethical Committee before the study began (Approval No: FMBSUREC/01022022/Ali). Participants were recruited from March 2022 to November 2024, and all follow-up evaluations were completed by the end of September 2024. The trial followed the CONSORT 2010 guidelines for reporting randomized trials. The process of patient enrollment, randomization, intervention allocation, and follow-up is illustrated in the CONSORT flow diagram [Figure 1] and the completed CONSORT checklist is provided as Supplementary File 1. No patients or members of the public were involved in the design, conduct, or reporting of this study.

### Inclusion and exclusion criteria

Children aged 3 to 14 years with single renal pelvic stone < 2 cm with density > 1000 HU were eligible for inclusion. Patients were excluded if they had a history of prior renal surgery, active urinary tract infection, distal obstruction, congenital renal anomalies, coagulopathy, or renal insufficiency.

### Sample size calculation

The sample size was calculated using G*Power version 3.1 software. Based on findings from a pilot study including 10 pediatric patients (5 in each group), the observed stone-free rate was 90% for the RIRS group and 95% for the mini-PCNL group. These values (P1 = 0.90 and P2 = 0.95) were used for the calculation of the required sample size for comparison of two proportions. Setting a two-tailed α error of 0.05 and a study power (1–β) of 80%, the minimum required total sample size was 58 patients (29 in each group). To compensate for possible losses during follow-up, the final sample was increased to 70 patients (35 in each group).

### Outcomes

The primary outcome was the stone-free rate at 1 month, defined as complete clearance or only clinically insignificant fragments ≤ 2 mm) on low dose NCCT. Secondary outcomes included operative time, hospital stay, catheterization duration, hemoglobin drop, radiation exposure, and perioperative complications classified by the modified Clavien–Dindo system. No interim analyses or stopping guidelines were planned.

### Preoperative assessment

All patients were evaluated preoperatively through history taking, physical examination, laboratory investigations (including complete blood count, coagulation profile, renal function tests, urine culture when indicated), and radiological imaging (KUB X-ray, ultrasound, and NCCT). Low-dose NCCT was performed preoperatively to confirm stone density and anatomy in accordance with ALARA principles to minimize radiation exposure. Informed written consent was obtained from the parents of all participants after full explanation of the procedure and follow-up plan.

#### Randomization and blindness

Patients were randomly assigned to one of the two groups using computer-based random number generation with sequentially numbered, sealed opaque envelopes by independent research staff.

The randomization sequence was generated by the research team using a validated computer algorithm, ensuring allocation concealment and minimizing selection bias. Envelopes were opened only after patient enrollment to assign the intervention.

Blinding was applied at two levels: patients and postoperative outcome assessors were blinded to group allocation. Surgeons could not be blinded due to the nature of the interventions; however, they were not involved in postoperative imaging interpretation or statistical analysis. Postoperative NCCT images were reviewed by a radiologist blinded to treatment assignment.

Complete blinding was not feasible due to the visible flank puncture and dressing associated with mini-PCNL. To minimize detection bias, postoperative outcomes were assessed using predefined objective criteria, and radiologic evaluation of stone-free status was performed by an independent radiologist who was not involved in surgical management or postoperative care.

This approach minimized detection and assessment bias while maintaining ethical and practical feasibility. All operations were performed by the same surgical team experienced in pediatric endourology to minimize inter-operator variability. Both interventions were performed under general anesthesia, and postoperative care protocols were identical to maintain participant blinding.

### Surgical techniques

Procedures were performed only at the Department of Urology by surgeons experienced in pediatric endourology. All interventions were performed as per protocol with full adherence; no crossover occurred.

### Mini-PCNL-Group

Under general anesthesia, retrograde ureteral catheterization was first performed in the lithotomy position using a 4 Fr ureteral catheter. With the patient in the prone position, the pelvicalyceal system was opacified via contrast injection. Percutaneous access was obtained under fluoroscopic guidance with an 18-G needle. Tract dilation was done using Amplatz dilators up to 16 Fr, and access sheaths were inserted over a 0.035-inch guidewire. Stone fragmentation was performed using a pneumatic lithotripter through 12 Fr rigid pediatric nephroscope (Karl Storz). Pneumatic lithotripsy was selected as the standard fragmentation method for rigid mini-PCNL due to its proven efficiency and minimal thermal risk. In future studies, incorporation of suction-irrigation systems or laser lithotripsy for mini-PCNL may improve comparability between techniques and potentially reduce operative time. Pneumatic lithotripsy was selected as the standard fragmentation method for rigid mini-PCNL due to its proven efficiency and minimal thermal risk. Fragments were extracted using stone forceps. A double-J stent was retained for four weeks to ensure drainage and prevent postoperative ureteral edema; no cases of stent encrustation were encountered.

### RIRS-Group

All RIRS patients underwent preoperative DJ stent placement two weeks prior to definitive surgery. This staged approach adds an additional procedure, anesthesia exposure, and hospital visit compared to mini-PCNL, which must be considered when assessing the total treatment burden. Under general anesthesia in the lithotomy position, a 0.035-inch safety guidewire was inserted. A 9.5/11.5 Fr ureteral access sheath (Boston Scientific) was advanced over the wire. Flexible ureteroscopy was performed using a 9.5 Fr LithoVue scope (Boston Scientific). Stone dusting was achieved using a Holmium: YAG laser (200 μm fiber, 100 W) with energy settings of 0.4–0.6 J at 15–20 Hz. Holmium: YAG laser was used because of its ability to achieve fine dusting with minimal retropulsion.Saline irrigation was maintained via syringe to ensure a clear visual field. A postoperative DJ stent was left in place for 4 weeks.

### Postoperative assessment

Postoperative parameters included operative time, radiation exposure time, hemoglobin drop, catheter duration, hospital stay, and complications. All complications were recorded and classified according to the modified Clavien–Dindo grading system. A urethral catheter was placed postoperatively to monitor urine output and was removed before discharge. After one month, NCCT was performed to evaluate stone clearance. Low-dose NCCT was used only once postoperatively, following ALARA guidelines. Patients were considered stone-free if no residual stones or fragments ≤ 2 mm were present. All peri-operative complications were prospectively recorded and graded according to the modified Clavien–Dindo classification. No additional or concomitant procedures were performed during follow-up.

### Statistical analysis

Data were analyzed using IBM SPSS Statistics for Windows, Version 25.0 (IBM Corp., Armonk, NY). Normality of distribution was verified using the Kolmogorov–Smirnov test. Continuous variables (e.g., operative time, hospital stay, catheterization duration, hemoglobin levels, radiation exposure) were expressed as mean ± standard deviation and compared between groups using independent samples t-tests. Within-group changes in hemoglobin levels were assessed using paired samples t-tests. Between-group hemoglobin drop was analyzed using independent t-tests with 95% confidence intervals. Categorical data (e.g., stone-free rate, complication grades) were expressed as frequencies and percentages and compared using chi-square tests. A p-value < 0.05 was considered statistically significant. All randomized participants were included in the final analysis.

## Results

This study (Table 1) comprised 70 pediatric patients with renal pelvic stone less than two centimeters in size. Study participants were randomly allocated into two equal groups. One group of 35 patients underwent RIRS, while the other group was treated with mini-PCNL. The two groups were similar regarding age, gender, stone size, stone density, and other baseline characteristics. No statistically significant differences were noted (*p* > 0.05).

**Table 1:** Sociodemographic characteristics of RIRS and Mini-PCNL groups

Characteristic	RIRS Group (*n* = 35)	Mini-PCNL Group (*n* = 35)	*p*-value
Preoperative Data			
Age (years, Mean ± SD)	7.89 ± 2.13	7.63 ± 2.50	0.644
Sex (Male/Female)	25/10	26/9	0.788
Stone Size (mm, Mean ± SD)	14.74 ± 3.20	14.63 ± 3.15	0.881
Stone Density (HU, Mean ± SD)	1236.71 ± 118.64	1202.74 ± 98.60	0.197
Preoperative Hemoglobin (g/dl, Mean ± SD)	12.75 ± 1.59	12.96 ± 1.67	0.585
Postoperative Hemoglobin (g/dl, Mean ± SD)	12.69 ± 1.59	12.22 ± 1.68	0.233

In Table 2 the mean operative time for the RIRS group was significantly shorter when compared to the mini-PCNL group (72.69 ± 8.96 min vs. 80.46 ± 8.18 min; *p* < 0.001). In line with the previous finding, the duration of hospital stay was also significantly shorter within the RIRS group (1.09 ± 0.28 days) compared to the mini-PCNL group (1.29 ± 0.46 days; *p* = 0.032). Mean catheterization time was shorter as well in the RIRS group (1.17 ± 0.38 days) compared to the mini-PCNL group (2.11 ± 0.40 days; *p* < 0.001). The RIRS group also had a significantly lower radiation exposure time compared to the mini-PCNL group (34.60 ± 4.35 s vs. 115.14 ± 21.06 s; *p* < 0.001).

**Table 2:** Safety comparison between RIRS and Mini-PCNL groups

Parameter	RIRS Group (*n* = 35)	Mini-PCNL Group (*n* = 35)	*p*-value
Preoperative DJ Stent Placement			
Patients with DJ stent	35 (100%)	0 (0%)	< 0.001
Number of procedures prior to definitive surgery	1	0	< 0.001
Additional anesthesia exposure (procedures)	1	0	< 0.001
Hospital visits prior to definitive surgery	1	0	< 0.001
Definitive Surgery Data			
Operative Time (min, Mean ± SD)	72.69 ± 8.96	80.46 ± 8.18	< 0.001
Radiation Exposure Time (sec, Mean ± SD)	34.60 ± 4.35	115.14 ± 21.06	< 0.001
Catheterization Time (days, Mean ± SD)	1.17 ± 0.38	2.11 ± 0.40	< 0.001
Postoperative Parameters			
Hospital Stay (days, Mean ± SD)	1.09 ± 0.28	1.29 ± 0.46	0.032
Hemoglobin Changes			
Preoperative Hemoglobin (g/dL, Mean ± SD)	12.75 ± 1.59	12.96 ± 1.67	0.585
Postoperative Hemoglobin (g/dL, Mean ± SD)	12.69 ± 1.59	12.22 ± 1.68	0.233
Hemoglobin Drop (g/dL, Mean ± SD)	0.01 ± 0.05 (*p* = 0.097)	0.74 ± 0.25 (*p* < 0.001)	< 0.001
Stent Duration			
Postoperative DJ Stent Duration (weeks)	4	4	—
Total DJ Stenting Period (weeks)	6	4	—

*Within-group p-values are based on paired samples t-tests: RIRS Group: t(34) = 1.706, *p* = 0.097, 95% CI [− 0.00284, 0.03256], Mini-PCNL Group: t(34) = 17.173, *p* < 0.001, 95% CI [0.656, 0.829], **Between-group p-value is based on an independent samples t-test:t(68) = 15.695, *p* < 0.001, 95% CI [0.599, 0.773], Hemoglobin Drop Calculation: Hemoglobin drop (ΔHb) was calculated by subtracting the post-operative hemoglobin value from the pre-operative hemoglobin value for each patient (ΔHb = HBpre - HBpost)

Concerning hemoglobin levels, the average decrease of hemoglobin concentration in the RIRS group was negligible and nonsignificant (0.01486 ± 0.05153 g/dL; *p* = 0.097). However, for the mini-PCNL group, the decrease was considerably larger (0.74 ± 0.25 g/dL; *p* < 0.001). The between group difference of hemoglobin decrease was significant (*p* < 0.001).

Table 3 shows that the stone-free rate (SFR) at one-month post-operative follow-up was 91.4% in the RIRS group and 94.3% in the mini-PCNL group, showing no significant difference between groups (*p* = 0.643). Complications occurred in 4 (11.4%) RIRS cases and 6 (17.1%) mini-PCNL cases, mostly minor (Grade I–II). Most complications were mild and included, mild hematuria, stent-related discomfort or transient fever; no major (Grade ≥ III) complications were reported. There was no statistically significant difference between the two groups (*p* = 0.47).

**Table 3:** Efficacy comparison at 1-Month Follow-Up: stone clearance and complication profile according to Clavien-Dindo classification

Outcome (1-Month Follow-up)	RIRS (*n* = 35)	Mini-PCNL (*n* = 35)	*p*-value
Stone Clearance			
Complete Clearance or residual ≤ 2 mm	32 (91.4%)	33 (94.3%)	0.643
Residual > 2 mm or Complete Failure	3 (8.6%)	2 (5.7%)	0.643
**Complication Profile (Clavien-Dindo Classification)**			
Grade I Stent-related discomfort Transient hematuria			0.47
	2 (5.7%)	3 (8.6%)	
	1 (2.9%)	2 (5.7%)	
Grade II Fever	6 (17.1%)	8 (22.9%)	
	1 (2.9%)	1 (2.9%)	

Success: Complete Clearance or residual ≤ 2 mm on 1-month imaging

Failure: Residual >2mm or Complete Failure

Complications categorized according to Clavien-Dindo Classification

## Discussion

The current research confirms that retrograde intrarenal surgery (RIRS) and mini-percutaneous nephrolithotomy (mini-PCNL) remain effective and safe techniques for the management of renal stones less than 2 cm in pediatric patients. Although both techniques RIRS and mini-PCNL have their merits, RIRS does indeed show some advantages, particularly in the perioperative factors. For example, RIRS required a significantly less surgery time (72.7 ± 9.0 min vs. 80.5 ± 8.2 min, *p* < 0.001), reduced exposure to radiation (34.6 ± 4.4s vs. 115.1 ± 21.1s, *p* < 0.001), shortened length of stay (1.09 ± 0.28 vs. 1.29 ± 0.46 days, *p* = 0.032), and demonstrated a markedly smaller reduction in hemoglobin level (0.015 ± 0.052 g/dL vs. 0.74 ± 0.25 g/dL, *p* < 0.001). These findings highlight RIRS as a less invasive yet equally effective alternative for selected pediatric cases.

### Clinical significance of perioperative differences

While the absolute differences in operative time and hospital stay were statistically significant, they were relatively small (operative time difference < 10 min, hospital stay difference < 0.2 days). However, these small reductions have clinically meaningful implications in pediatric practice: (1) reduced anesthesia exposure minimizes potential complications in pediatric patients; (2) faster recovery times reduce the psychological and physical impact on children and families; (3) reduced postoperative monitoring needs; and (4) decreased overall cost burden. The substantial difference in hemoglobin drop (0.01 vs. 0.74 g/dL) reflects the more invasive nature of mini-PCNL and has important implications for patient safety and recovery.

Although some statistically significant differences demonstrated limited absolute magnitude, these findings should be interpreted within the pediatric context. Even modest reductions in operative time and hospital stay may be clinically meaningful in children, as they translate into reduced anesthesia exposure, shorter hospitalization, and lower overall treatment burden for patients and caregivers.

### Comparative literature review

Several relevant studies confirmed the reported results. Similar findings were reported by Mahmoud et al. [10] where RIRS was found to be associated with decreased fluoroscopy time and reduced length of stay without any significant increase in complications or stone-free rates when compared to mini-PCNL. The latter authors, Resorlu et al. [11], Pelit et al. [12], all noted stone clearance rates were similar between the two techniques, but RIRS was often associated with a more favorable perioperative course and lower morbidity.

Baş et al. [13] observed no statistically significant differences in the success and complication rates of RIRS and micro-PCNL in RIRS children with renal stones measuring 10–20 mm. However, they confirmed that the RIRS group had a significantly lower length of hospital stay and radiation exposure, which corroborates our findings. Soliman Othman et al. [14] also noted greater operative efficacy and less need for retreatment in F-URS relative to mini-PCNL and ESWL for stone sizes 10–20 mm.

Jia et al. [15] reported comparable stone-free rates between flexible ureteroscopy and super-mini PCNL, with RIRS having a clear advantage due to the lower operative time and length of hospital stay, as well as lower drop in hemoglobin levels. Akbulut et al. [16] further emphasized that while lower calyceal stones might be cleared more effectively with mini-PCNL, the recovery profile in terms of complications is significantly better with RIRS.

Saad et al. [17] studied the > 2 cm stones and noted that although PCNL had a better SFR (95.5% vs. 71%), it was associated with higher complication rates and longer hospital stays. While this investigation is centered around smaller stones (< 2 cm), these outcomes align with the expectation of enhanced RIRS safety and recovery.

Elsheemy et al. [18] highlighted that mini-PCNL was more effective than ESWL in preschool children with lower calyceal stones, which illustrates the necessity of endoscopic procedures, suggesting the use of RIRS or mini-PCNL in more complex or geographically difficult surgical areas.

As mentioned by Elmoguy et al. [19], all three techniques F-URS, mini-PCNL, and ESWL were considered safe; however, mini-PCNL did have the longest operative and fluoroscopy times. This perspective allows us to further reinforce the argument that RIRS is positioned safely in the spectrum of intervention within the triad of safety, effectiveness, and invasiveness.

Taken together, the available evidence strongly supports the use of RIRS as the first approach for pediatric patients with renal stones < 2 cm in diameter, especially when considering anatomical access. The option of mini-PCNL should be preserved for cases where RIRS is limited technically or in patients with a greater stone burden situated in the lower calyceal regions.

### Study limitations

Several limitations should be acknowledged. Routine preoperative double-J stenting in the RIRS group, although intended to enhance procedural safety in pediatric patients, represents an additional intervention with extra anesthesia exposure and hospital visits, potentially increasing treatment burden and introducing imbalance when the entire treatment pathway is considered. This study was conducted at a single center with a relatively small sample size, which may limit generalizability. The short follow-up period precluded assessment of long-term outcomes such as stone recurrence and late renal functional changes. Differences in lithotripsy modalities between groups (Holmium: YAG laser for RIRS versus pneumatic lithotripsy for mini-PCNL) may have influenced operative efficiency and represent a potential confounding factor. Although primary RIRS was feasible in 100% of carefully selected patients in our cohort, real-world applicability may be affected by variability in patient compliance with staged procedures, anatomical suitability, and follow-up reliability, underscoring the importance of individualized patient selection in pediatric practice. Finally, surgeon blinding was not feasible due to the percutaneous nature of mini-PCNL; however, objective outcome measures and independent radiologic assessment were used to minimize detection bias, and statistically significant differences with small absolute magnitude should be interpreted cautiously within the pediatric clinical context.

## Conclusion

Both RIRS and mini-PCNL are effective and safe options for managing pediatric renal pelvic stones < 2 cm with density more than 1000 HU. In this randomized study, both modalities achieved comparable stone-free rates with acceptable safety profiles. However, RIRS demonstrated more favorable perioperative outcomes, including shorter operative time, reduced radiation exposure, smaller hemoglobin drop, and shorter hospital stay. These findings emphasize the minimally invasive nature and faster recovery associated with RIRS, rather than a true superiority in efficacy. It is important to acknowledge that all RIRS patients required preoperative DJ stent placement, adding an additional procedure and hospital visit. Therefore, when considering the entire treatment pathway, clinicians should weigh the perioperative advantages of RIRS against the additional preoperative procedural burden. While mini-PCNL remains a valid alternative, particularly in cases of anatomical complexity or lower calyceal burden, RIRS can be considered a preferred first-line option in carefully selected pediatric patients with appropriate follow-up capabilities. Further multicenter studies with larger samples, longer follow-up periods, and standardized surgical techniques are warranted to confirm these results and evaluate long-term renal outcomes.

## Supplementary Information

Below is the link to the electronic supplementary material.

Supplementary Material 1

## Data Availability

The datasets used and/or analyzed during the current study are available from the corresponding author on reasonable request.
